# How to Make a Cost Model for the Birth Cohort Biobank in China

**DOI:** 10.3389/fpubh.2020.00024

**Published:** 2020-02-21

**Authors:** Meiqin Wu, Deqing Wu, Chunping Hu, Chonghuai Yan

**Affiliations:** ^1^MOE, Shanghai Key Laboratory of Children's Environmental Health, Xinhua Hospital Affiliated to Shanghai Jiao Tong University School of Medicine, Shanghai, China; ^2^Department of Gastroenterology, Shanghai Tenth People's Hospital, Tongji University School of Medicine, Shanghai, China; ^3^School of Public Health, Shanghai Jiao Tong University, Shanghai, China

**Keywords:** biological specimen bank, budget, cohort study, cost analysis, sustainability

## Abstract

Biobanks and cohort studies are a popular topic in China these days and even in the global scientific research field. Cohorts with biological material are necessary to investigate potential biological mechanisms behind a disease and its early detection. Establishing a biobank is expensive and the long-term sustainability of biorepositories is a key issue globally. There is some published information on tools to calculate the biospecimen user fee; however, they may not be suitable for China's biobanks (as most of the biobanks in China are not for profit and are funded by government or research grants, and as such, funding is a major constraint). The limited published data also tend to be highly variable and specific to the type of biobank. The authors of this article aim to present the basis of a cost analysis model for a biobank of human biological samples of a birth cohort in Shanghai, China. The results show that it is very practical for us to consider how to build a cost model for the birth cohort biobank from the direct funds, such as storage equipment, temperature monitoring system, information management system, and so on. We conclude that by comparing the similarities and differences between China's cost model and that of other countries, this paper provides valuable information for biobankers to identify new perspectives on potential collaborators and mutual learning opportunities.

## Introduction

Today, biobanks are playing increasingly important roles in clinical and translational research, especially in the area of cohort study. A cohort study is a long-term research strategy involving a biobank, and comprises the collection, processing, storage, and dissemination of systematically organized biological samples and their associated clinical data, all of which are critical prerequisites for high quality biomedical research (1). The UK biobank, launched in 2007, targets half a million adult Britons (2), and similarly large cohorts are being enlisted in Canada (3), Norway (4), Netherlands (5), Denmark (6), Ireland (7), the United States (8), and so on. These biobanks represent a precious resource for health research, especially in view of the fact that newly constructed biobanks will take many years to accumulate enough cases of chronic diseases in their prospective cohorts to allow relevant biomarker research (9). It will have significant consequences in determining both the opportunities for and the limitations of future research into the genetic and environmental contributors to complex diseases (10). Similar to the rapid development of biobanks worldwide, biobanks have been proliferating in China and making great progress over the years (11). However, there are many differences between the construction and management of China's biobanks and those of other countries. Most of the biobanks in China are not for profit and are funded by government or research grants—as such, fund shortage is a crucial bottleneck problem in the development of China's biobanks (12). Thus, developing sustainability models for biobanks is essential, as a significant amount of funds is required to run a biobank on an annual basis.

However, in the international scientific literature, there are no proposals on specific cost accounting models for these types of organizations (13). A biospecimen user fee calculator tool developed by the Canadian Tissue Repository Network (CTRNet), a tumor biobank network, was published in 2014 and is accessible online at www.biobanking.org (14). Additionally, the National Institutes of Health National Cancer Institute's Biorepositories and Biospecimen Research Branch has developed the Biobank Economic Modeling Tool (BEMT), which will be able to create a cost profile for their biobanks' specimens, products, and services, as well as establish pricing and allocate costs for biospecimens (15). Although this tool is very good, it may not be suitable for Chinese biobanks. The development of a unique cost model for biobanks can be a complicated task because of the diversity of the biological samples they store. Thus far, nothing has been published on China's biobank construction budget.

Therefore, the aim of this paper is to present the basis of a cost analysis model used for the biobank which support the collection, analyses, storage and distribution of biospecimens for the Shanghai Birth Cohort Study (SBC, www.shyousheng.net), which is a prospective birth cohort that enrolls pregnant women, fathers to-be, and newborns from nine participating hospitals throughout Shanghai over 2013–2015 (16). This information may be of interest to groups considering establishing or that are already in the process of establishing biospecimen repositories.

## Materials and Methods

### SBC Biobank and SBC Biobank Activities Evaluated

**Figure 1:**
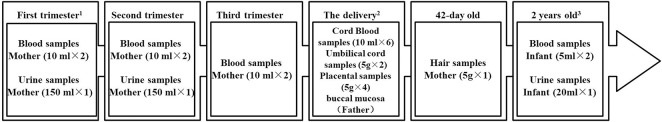
Sample collection in the SBC biobank. 1. Two of nine participating hospitals recruit candidates from the pre-pregnant period, which have pre-pregnancy outpatient service. 2. The fathers-to-be were asked to give the buccal mucosa samples at any time, but mainly at this time. And the spot blood samples of the newborns' were collected 3–7 days after birth. 3. This is the phase I plan. There will be continuous long-term follow-up for the participants.

The SBC biobank was launched in 2012 as an amendment to an ongoing research, the SBC (17). The overall aim of the SBC is to study the effects of environmental factors [e.g., perfluoroalkyl and polyfluoroalkyl substances (18), bisphenol A (19), triclosan, and pesticide (20) so on] on pregnancy outcomes and later health of the children as well as the parents. The study is designed to enroll 4,000 early gestation women, their husbands and newborns. Inclusion and exclusion criteria were described in detail in the published article “Cohort profile: the Shanghai Birth Cohort” (i) they were 20 years of age or older; (ii) at least one of the couple was a registered Shanghai resident; (iii) they planned to seek prenatal care and give birth at the SBC participating hospitals; (iv) the family intended to stay in the catchment area for at least 2 years; and (v) they were willing to sign a consent form and be followed for at least 2 years. Data are collected on the level of mother, father, and the child. Detailed information on demographics, environment, behavior, nutrition, sleep, and medical history of mother and child, will be recorded (17). The women are recruited at the initial prenatal care visit during the preconception stage or first trimester (P1, mother's blood and urine are collected). Regular follow-up assessments are conducted when they visit the research office for the second consultation at 24–28 weeks of gestation (P2, mother's blood and urine are collected). At 32–36 weeks of gestation, women visit for the third consultation (P3, mother's blood is collected). During delivery, women's medical records are reviewed and abstracted, and umbilical cord blood, placental, umbilical cord, spot blood (3–7 days after birth), and the father's buccal mucosa are collected. As part of routine clinical care, postpartum women and infants are required to return to the delivery hospital for routine postpartum care 42 days after birth (mother's hairs are collected). After 6, 12, and 24 months, children are contacted by research assistants for follow-up visits, but only during the 2-year follow-up do the research assistants collect the blood and urine samples of children. The specific sample collection of each period is shown in Figure 1. The methods of SBC have already been described in detail elsewhere (17). This project was approved by the ethics committee of Xinhua Hospital affiliated to Shanghai Jiao Tong University School of Medical. At the time of recruitment, the couple signed the informed consent form. When the 2 years old follow-up, the informed consent needed sign again with the child's guardian (parents). The biological sample bank of Xinhua Hospital has passed the qualification of human genetic resources preservation issued by the Ministry of Science and Technology of the People's Republic of China, and it is consistent with the national biosafety and national defense rules.

### Implementation of the Cost Model

Before formally kicking off the SBC, we conducted a pilot study (21–23) to adjust the standard operating procedures (SOPs) for sample collection, loading, transportation, and storage and to determine the necessary equipment and consumables, which is conductive to the construction of the infrastructure of the biobank base at the same time. Table 1 shows the items that should be considered during the construction of the SBC biobank and in ensuring its sustainability. Some of them are funded by research grants, while others are funded by Xinhua Hospital (XH) and Shanghai Jiao Tong University (SJTU) School of Medicine. The SBC biobank was built on a project basis; therefore, the model presented in this study is limited to the budget for the portion of the grant to be paid for. The funding from Xinhua Hospital and Shanghai Jiao Tong University School of Medicine will not be included in our model.

**Table 1:** Cost considerations for setting up the SBC biobank.

**Category**	**Item**	**Funded by**
**FACILITIES**		
1. Building and power supply	Physical building	XH^a^
	Heating, ventilation and air conditioning	XH
	Lighting	XH
	Flooring	XH
	Backup power	XH
	Access control systems	XH
	Security cameras	XH
	Fire prevention systems	XH
	−20°C walk-in environmental storage systems	XH
2. Equipments^c^	Mechanical freezers (freezers, fridges, liquid nitrogen tanks)	Grants
	Stainless steel racks	Grants
	Freezer boxes	Grants
	Barcode printers	Grants
	Barcode scanners	Grants
	Temperature monitoring system	Grants
	Temperature monitors	Grants
	Information management system	Grants
	Computer equipment (hardware and software)	SJTU^b^
	Equipment for sample processing (centrifugal machines, biosafety cabinets)	SJTU
	Automatic nucleic acid extractor	SJTU
	Thermal cycler and Gel Doc	SJTU
	Sample quantization equipment	SJTU
	Autoclave	SJTU
	Water purification system	SJTU
**PERSONNEL**		
Staff	Manager, scientists, technician, data and IT manager	XH
Training	Equipment use, quality control and quality assurance practices, biohazard and chemical hazard training, etc.	XH
**MAINTENANCE**		
Building	Maintenance and repair cost	XH
Equipment	Annual services and recalibration	XH
Temperature monitoring	SMS charge	Grants
**OVERHEADS**		
	Telephone, internet and electricity access	XH
	Bio hazard waste removal and cleaning services	XH
**CONSUMABLES**		
1. Collection	Collecting tubes/vials/cups, needles, general label with barcode	Grants
2. Processing	Reagent, pipets, tips	Grants
3. Shipping	Transportation costs of cold chain	Grants
4. Storage	Plates/tubes/vials, freezer label with barcode	Grants
5. General	Other consumables including basic stationery and personal protection	Grants

a *XH, Xinhua Hospital*.

b *SJTU, Shanghai Jiao Tong University School of Medicine*.

c *The equipment investment will be calculated in detail in Table 3 below*.

Our model only includes the storage equipment, temperature monitoring system, information management system, and the consumables for collection, processing, and storage of the different samples at different times, which are noted as “Grants” in Table 1. The study does not include the costs for specimen dissemination or testing either, which are covered under another item in China's budget. We only listed the necessary equipment and consumables and calculated the amount of funding needed from the grants. All the costs in this paper is measured in Renminbi (RMB), and 1 US$ is about 6.9 in RMB.

## Results

### Design of the Cost Analysis Model

#### Aliquot Schemes for Various Sample Types

**Figure 2:**
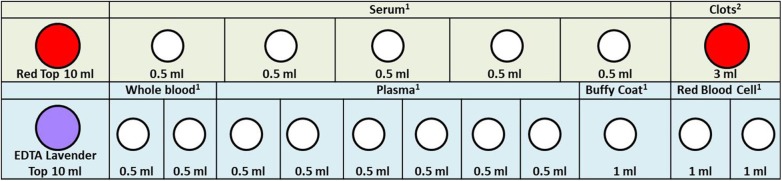
Maternal blood specimen processing rack diagram. 1. In order to facilitate the work of the sample assembler, we stored 0.5 ml serum, whole blood, plasma, and 1.0 ml buffy coat and red blood cell in the 1.2 ml frozen storage tubes. 2. Because the clots are not easy to take out, we stored them directly in the original tube. When we need to extract DNA, we take it out. The color of the acquisition and storage tube in the figure is set according to the color of its actual tube cover: red, 10 ml vacutainer-red top; lavender, 10 ml vacutainer-lavender top; white, cryogenic vials 1.2 ml.

**Figure 3:**
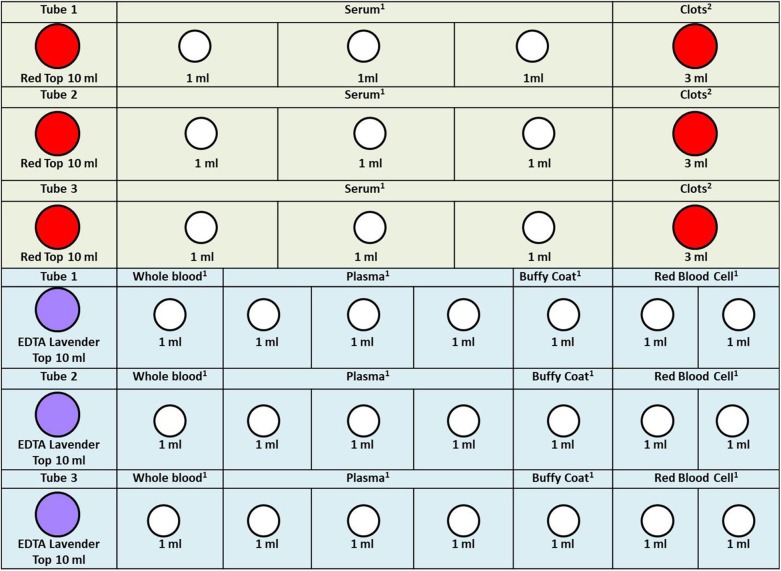
Cord blood specimen processing rack diagram. 1. In order to facilitate the work of the sample assembler, we stored 1.0 ml serum, whole blood, plasma, buffy coat, and red blood cell in the 1.2 ml frozen storage tubes. 2. Because the clots are not easy to take out, we stored them directly in the original tube. When we need to extract DNA, we take it out. The color of the acquisition and storage tube in the figure is set according to the color of its actual tube cover: red, 10 ml vacutainer-red top; lavender, 10 ml vacutainer-lavender top; white, cryogenic vials 1.2 ml.

**Figure 4:**
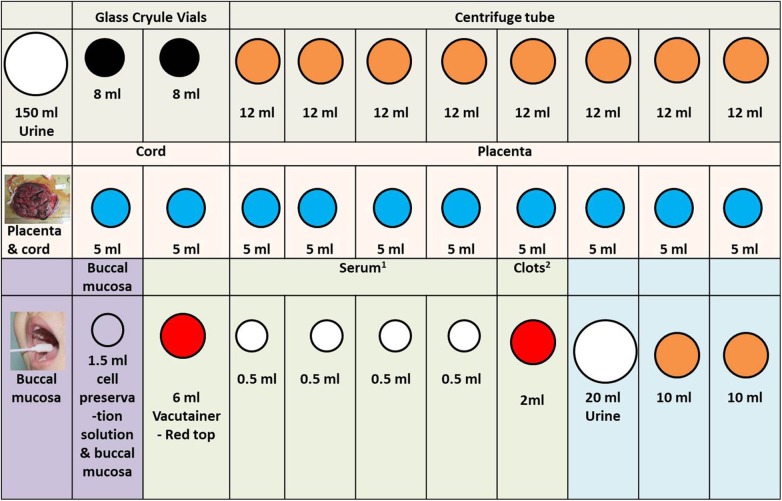
Other specimen in the SBC biobank processing rack diagram. 1. In order to facilitate the work of the sample assembler, we stored 0.5 ml serum in the 1.2 ml frozen storage tubes. 2. Because the clots are not easy to take out, we stored them directly in the original tube. When we need to extract DNA, we take it out. The color of the acquisition and storage tube in the figure is set according to the color of its actual tube cover: white (large), 200 mL urine collection cup; white (middle size), 1.5 ml cell preservation solution and buccal mucosa; white (small), cryogenic vials 1.2 ml; black, glass cryule vials 8 ml; orange, centrifuge tube; blue, cryogenic vials 5 ml; red, 6 ml vacutainer-red top.

In Figures 2–4, we determined the aliquot method according to the different sample types collected during each follow-up period in Figure 1, which is the basis for our next sample size calculation.

#### Freezing Methods

**Figure 5:**
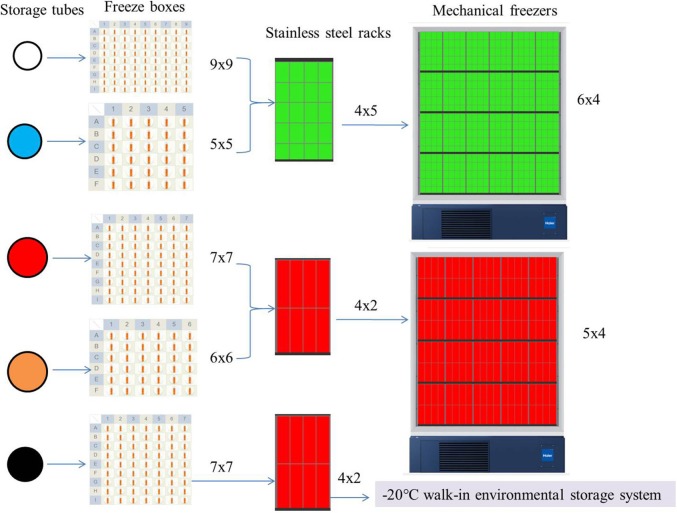
Freezing methods in the SBC biobank. The color of the storage tube in the figure is set according to the color of its actual tube cover: white 1.5 ml cell preservation solution and buccal mucosa and cryogenic vials 1.2 ml; blue, cryogenic vials 5 ml; red, 6 and 10 ml vacutainer-red top; orange, centrifuge tube; black, glass cryule vials 8 ml. The color of the stainless steel racks is virtual, it's actually all metallic gray.

The sub-packed samples were stored in different freeze boxes according to volume; then, the freeze boxes were placed on different stainless steel racks, and eventually uniformly stored in mechanical freezers and positioned according to the information management system (except for the urine, which was packed in a glass tube with a black cover, as it was stored in −20°C walk-in environmental storage systems). Figure 5 presents the freezing method used in the SBC biobank.

#### Sample Size and Freezer Space

Based on the above description and the sample collection in Figure 1, we can summarize and derive the sample size for each family according to the different sample types and different subpackage methods (Table 2). Storage space (the number of mechanical freezers to be purchased), freeze boxes, and racks involved in each follow-up period for the 4,000 families in the SBC biobank were also calculated in detail and shown in Table 2.

**Table 2:** Sample size and freezer space of the SBC biobank.

**Projects**	**Number of aliquots**					**Total**
	** 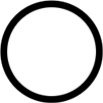 **	** 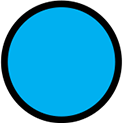 **	** 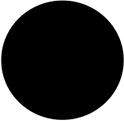 **	** 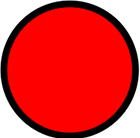 **	** 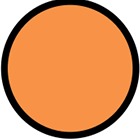 **	
P1^c^						
Blood	16	0	0	1	0	17
Urine	0	0	2	0	8	10
P2^c^						
Blood	16	0	0	1	0	17
Urine	0	0	2	0	8	10
P3^c^						
Blood	16	0	0	1	0	17
Delivery						
Blood	30	0	0	3	0	33
Placenta	0	8	0	0	0	8
Cord	0	2	0	0	0	2
Father's buccal mucosa	1	0	0	0	0	1
24-Month						
Blood	4	0	0	1	0	5
Urine	0	0	0	0	2	2
Sample size of each family^a^	83	10	4	7	18	122
Sample size of 4,000 families	332,000	40,000	16,000	28,000	72,000	488,000
Number of freezer boxes	4,099 (9 × 9)	1,600 (5 × 5)	327 (7 × 7)	571 (7 × 7)	2,000 (6 × 6)	8,597
Number of stainless steel racks	205 (4 × 5)	80 (4 × 5)	41 (4 × 2)	71 (4 × 2)	250 (4 × 2)	647
Number of mechanical freezers	8.54 (6 × 4)	3.33 (6 × 4)	–^b^	3.55 (5 × 4)	12.5 (5 × 4)	28

a *This sample size refers only to samples that require freezer boxes, stainless steel racks, or mechanical freezers. Filter paper (stored at 20°C walk-in environmental storage systems) which collected heel blood samples of newborns and mother's hair (stored at room temperature) which collected 42-day old were not included in this part, but included in the consumables investment*.

b *This part of the sample does not occupy the mechanical freezer space*.

c *P1, first trimester; P2, second trimester; P3, third trimester*.

#### Equipment Investment

Based on the eight items of equipment input funded by grant in Table 1 and the number of mechanical freezers calculated in Table 2, as well as the number of freeze boxes and stainless steel racks involved in this process, we first calculated the equipment investment (Table 3), which is 2,419,278 RMB–(A).

**Table 3:** Equipments investment of the SBC biobank.

**Item**	**Brand/Product ID**	**Unit-price**	**Amount needed**	**Total (RMB)**
Mechanical freezers (freezers, fridges, liquid nitrogen tanks)^a^	Haier/DW-86L728	55,000	31	1,705,000
Stainless steel racks	Youjia	360	647	232,920
Freezer boxes	Youjia	14	8,597	120,358
Barcode printers	Brady/IP 3000	23,000	3	69,000
Barcode scanners	Symbol/DS6708	1,000	8	8,000
Temperature monitoring system	Haier/U-COOL	free	1	0
Temperature monitors	Haier/U-COOL	3,000	28	84,000
Information management system	BIMS/Haier	200,000	1	200,000
Total (RMB)				2,419,278

a *According to the calculation in Table 2, the number of mechanical freezers is 28. But there should be 10% reserve refrigerators in accordance with the Isber guidelines, so the actual number of refrigerators purchased is 31*.

#### Consumables

Consumables need to be considered from the five aspects of sample collection, processing, shipping, storage, and general expenses according to Table 1 (shipping fees will be calculated in the next part). According to the different follow-up periods in Figure 1, we calculate the consumables investment of each family (Table 4), which is 286.68 RMB. According to the SBC project, the total consumables investment of 4,000 families is 286.68 × 4,000 = 1,146,720 RMB–(B).

**Table 4:** Consumables investment for each family of the SBC biobank.

**Visit time**	**Item**	**Brand/Product ID**	**Unit-price (RMB)**	**Amount needed**	**Total (RMB)**
P1^a^	21 gauge needle	BD/367287	0.20	1	0.20
	10 ml vacutainer-red top	BD/367820	1.00	1	1.00
	10 ml vacutainer-lavender top	BD/367525	1.00	1	1.00
	200 mL urine collection cup	JND/130835	3.50	1	3.50
	General label for collection container	BD/ Y3170707	0.27	3	0.81
	Cryogenic vials 1.2 ml (−80°C)	Nalgene/5000-1012	1.35	16	21.60
	Glass cryule vials 8 ml (−20°C)	CNW/VJEQ-38013-13100-100	3.85	2	7.70
	Centrifuge tube (−80°C)	Corning/ 430791	1.15	8	9.20
	10 ml serological pipet	Corning /4488	1.85	1	1.85
	Specimen/freezer label	BD/Y3076151	0.60	27	16.2
	General	–	1.50	1	1.50
P2^a^	21 gauge needle	BD/367287	0.20	1	0.20
	10 ml vacutainer-red top	BD/367820	1.00	1	1.00
	10 ml vacutainer-lavender top	BD/367525	1.00	1	1.00
	200 mL urine collection cup	JND/130835	3.50	1	3.50
	General label for collection container	BD/ Y3170707	0.27	3	0.81
	Cryogenic vials 1.2 ml (−80°C)	Nalgene/5000-1012	1.35	16	21.60
	Glass cryule vials 8 ml(−20°C)	CNW/VJEQ-38013-13100-100	3.85	2	7.70
	Centrifuge tube (−80°C)	Corning/ 430791	1.15	8	9.20
	10 ml serological pipet	Corning /4488	1.85	1	1.85
	Specimen/freezer label	BD/Y3076151	0.60	27	16.20
	General	–	1.50	1	1.50
P3^a^	21 gauge needle	BD/367287	0.20	1	0.20
	10 ml vacutainer-red top	BD/367820	1.00		1.00
	10 ml vacutainer-lavender top	BD/367525	1.00	1	1.00
	General label for collection container	BD/Y3170707	0.27	2	0.54
	Cryogenic vials 1.2 ml (−80°C)	Nalgene/5000-1012	1.35	16	21.60
	Specimen/freezer Label	BD/Y3076151	0.60	17	10.20
	General	–	1.00	1	1.00
Delivery	21 gauge needle	BD/367287	0.20	1	0.20
	10 ml vacutainer-red top	BD/367820	1.00	3	3.00
	10 ml vacutainer-lavender top	BD/367525	1.00	3	3.00
	Cryogenic vials 1.2 ml (−80°C)	Nalgene/5000-1012	1.35	30	40.50
	Cryogenic vials 5 ml (−80°C)	Youjia YJ5	1.00	10	10.00
	903 protein saver snap-apart card	Whatman	2.50	1	2.50
	Oral swabs	Fenglin	13.00	1	13.00
	General label for collection container	BD Y3170707	0.27	10	2.70
	Specimen/freezer label	BD/Y3076151	0.60	45	27.00
	General	–	2.50	1	2.50
42-day	Zipper bag	Apple 3#	0.06	1	0.06
	Specimen/freezer label	BD Y3076151	0.60	1	0.60
	General label for collection container	BD Y3170707	0.27	1	0.27
	General	–	0.50	1	0.50
24-month	21 gauge needle	BD367287	0.20	1	0.20
	6 ml Vacutainer-red top	BD367820	1.00	1	1.00
	200 mL urine collection cup	JND130835	3.50	1	3.50
	Centrifuge tube(−80°C)	Corning 430791	1.15	2	2.30
	General label for collection container	BD Y3170707	0.27	2	0.54
	Cryogenic vials 1.2 ml (−80°C)	Nalgene 5000-1012	1.35	3	4.05
	Specimen/freezer label	BD Y3076151	0.60	6	3.60
	General	–	1.00	1	1.00
Total (RMB)					286.68

a *P1, first trimester; P2, second trimester; P3, third trimester*.

#### SMS Charge and Transportation Costs of Cold Chain

In China, short message service (SMS) charges have the lowest monthly rental fee and vary according to the package used. It is estimated that 100 RMB per month should be enough for the SBC biobank. The project takes about 5 years; therefore, SMS charges = 100 × 12 × 5 = 6,000 RMB–(C).

Cold chains are frequently used in a biobank, especially in multicenter studies. At present, there is no unified national standard for this aspect of the charges in China. Most of the charges will vary according to the distance, the sample size of each transport, the means of transportation, and so on. The SBC biobank sample consists of nine hospitals in Shanghai, with the exception of one hospital where the SBC biobank is located. Samples are shipped in freezer boxes; therefore, our cost is estimated based on the unit price per box. From Table 2, the number of common freezer boxes is 8,597 (small: 4,099 + 1,600 = 5,699; large: 327 + 571 + 2000 = 2,898). Apart from about 500 families in the hospital where the sample bank is located, there are 3,500 families, or 87.5% of the samples that need to be transported from the outside hospitals to the SBC biobank. The unit price of the small freezer box (9 × 9, 5 × 5) is 5 RMB while that of the large freezer box (7 × 7, 6 × 6) is 10 RMB. Thus, the transportation costs of cold chain: 5 × 5,699 × 87.5% + 10 × 2,898 × 87.5% = 24933.125 + 25357.5 = 50,290.63 RMB–(D).

#### Total Budget in the Model

According to the cost considerations in setting up the SBC biobank in Table 1, we have calculated the cost from the grant section, which includes equipment investment, consumables, SMS charges, and transportation costs related to the cold supply chain: (A) + (B) + (C) + (D) = 2,419,278 + 1,146,720 + 6,000 + 50290.63 = 3,622,288.63 RMB.

## Discussion

### Main Findings

Financial sustainability is a challenge that biobankers generally face. Thus, gaining access to funds is crucial in the sustainable development of a biobank. This is the first study to describe the budgetary model of China's biobank in the early stage of the birth cohort study. It will show readers the details to be considered and the differences between China and other countries in the process of building a biobank.

### Possible Differences Between China's Cost Model and That of Other Countries

Biorepositories are costly with respect to staffing, equipment, service contracts, consumables, and expertise (24). In the establishment and management of biobanks, there are many differences between China and other countries: (i) The staff of a biobank in China are usually employed by hospitals or schools, but seldom employed by the director of the biobank or the director of the laboratory (12). This means that the staffs' labor costs are paid by the hospital or the school, and not by the director of the biobank using the construction funds. Thus, the staff of the SBC Biobank, which includes the directors, scientists, technicians, data and IT managers and so on, are employed by Xinhua Hospital, which will then pay the wages of these employees. The training cost of the staff in the biobank is also paid by Xinhua Hospital. (ii) In China, hospitals or schools are responsible for the building, power supply, and purchase of fixed instruments and equipment. Thus, the biobanks themselves do not need to pay for them. (iii) The cost of maintenance of the building and equipment, telephone, internet, and electricity access, bio-hazard waste removal and cleaning services is undertaken by Xinhua Hospital, and not taken from the construction funds of the biobank. Thus, we do not include this part of the budget expenditure in our model. Therefore, our actual input is much more than that calculated in our model, and not just about 3.6 million RMB.

### Decide the Brand of Consumables and Sample Size According to the Amount of Budget Input

Collection and long-term storage of biological specimens are becoming integral parts of the cohort study (25). With the growth of biobanks and cross-border collaborations, there is also a need for greater international harmonization of regulation. The SBC has made many references to the National Children's Study (NCS) project, which was led by the National Institutes of Health and planned to recruit participants from across the United States of America from before birth until age 21 (26, 27). For example, the blood collection tubes (BD367820, BD367525) and blood freezing tube (Nalgene 5000-1012) used in the SBC were the same as those used in the NCS project (Table 3).

Compared to other animal experiments or basic research, cohort study requires much more capital investment. According to pre-experiment and project investment funds in the SBC biobank, the sample collection, preservation, and other processes were modified and re-planned. In addition to the follow-up points in Figure 1, the children in the SBC were to be followed up with after 6 and 12 months by telephone call, but there was no sample collection considering the budget and storage space limitations. Hence, these two points were not included in our cost model.

### Importance of Budgeting and Design for International Cooperation

As a big city in China, Shanghai has its advantages and disadvantages in the field of medical and health care. In order to properly make use of limited funds, the project team focused on many details of international integration and conducted a pilot study before the formal start of the SBC. Now, international cooperation has already been well-established with the University of Aarhus in Denmark (28) and we have also become one of the members of the Birth Cohort Consortium of Asia (29). At the same time, the SBC biobank takes part in the Maelstrom Research by the Research Institute of the McGill University Health Centre (RI MUHC) (30), which brings together an international team of epidemiologists, statisticians, and computer scientists to find practical solutions to some of the challenges faced by epidemiological research collaborations.

### Strengths and Limitations

Detailed records and calculations are the strengths of the present study. However, the design and development of detailed biobanking procedures in the field of birth cohort are currently lacking. Therefore, we describe the whole process of building a biobank without any foundation from a new point of view and summarize the aspects that a biobank director or project designer should consider in terms of space, time, population, and so on. High quality biological samples are a powerful guarantee for subsequent analysis and testing. However, they also have some limitations. First, this model may only be applicable to China's related cohort studies rather than to the rest of the world's. Second, the total amount is only calculated on the basis of the sample size of this project, which only serves as a reference for other projects' budget. Each project may require different inputs due to different sample sizes, sample types and follow-up frequencies.

## Conclusion

Cost analysis tools have become instrumental in ensuring budget optimization. This study has built a cost model for the birth cohort biobank in China. At the same time, by comparing the similarities and differences between China's cost model and that of other countries, this paper provides valuable information for biobankers looking forward to identify new perspectives on potential collaborators and mutual learning opportunities.

## Data Availability Statement

The datasets generated for this study are available on request to the corresponding author.

## Ethics Statement

The studies involving human participants were reviewed and approved by the Ethics Committee of Xinhua Hospital Affiliated to Shanghai JiaoTong University School of Medical. Written informed consent to participate in this study was provided by the participants' legal guardian/next of kin.

## Author Contributions

MW, DW, CH, and CY contributed to the study design and acquisition of research data. MW and DW conducted the data analysis. MW drafted the manuscript. All authors contributed to the improvement of the manuscript and approved the final version for publication.

### Conflict of Interest

The authors declare that the research was conducted in the absence of any commercial or financial relationships that could be construed as a potential conflict of interest.
